# Years of potential life lost due to drug-susceptible pulmonary tuberculosis in Cali, Colombia, 2009-2020

**DOI:** 10.17843/rpmesp.2025.423.14207

**Published:** 2025-09-08

**Authors:** Adriana M. Rivas-Mina, Jose F. Fuertes-Bucheli, Jennifer Lozano-García, Laura D. Luna, Yessenia Niño-Ramiréz, Lucy Luna, Robinson Pacheco López

**Affiliations:** 1 Grupo Interdisciplinario de Epidemiología y Salud Pública, Universidad Libre, Cali, Colombia. Universidad Libre Grupo Interdisciplinario de Epidemiología y Salud Pública Universidad Libre Cali Colombia; 2 Facultad de Ciencias de la Salud, Universidad Icesi, Cali, Colombia. Universidad Icesi Facultad de Ciencias de la Salud Universidad Icesi Cali Colombia; 3 Secretaría Distrital de Santiago de Cali, Cali, Colombia. Secretaría Distrital de Santiago de Cali Cali Colombia

**Keywords:** Life expectancy, pulmonary tuberculosis, mortality, premature mortality, health equity

## Abstract

A cross-sectional descriptive and analytical study evaluated years of potential life lost (YPLL) due to drug-susceptible pulmonary tuberculosis in Cali from 2009 to 2020, using death records from the Health Secretariat. Total YPLL and its annual rate were calculated by age at death, and median YPLL values were compared across subgroups. A total of 565 registers of death records were analyzed (71.5% men; median age 56 years, range 38-70), yielding 11,238 YPLL. The highest median YPLL were observed among persons living with HIV, those in socially vulnerable situations, the uninsured or those under subsidized or special coverage, in women, and in those who re-entered after previously discontinuing treatment or experiencing a tuberculosis relapse.

## INTRODUCTION

Tuberculosis (TB) is the leading cause of death attributable to an infectious agent, with >1 million deaths annually. Between 2015-2023, mortality decreased by 23% (far from the 75% reduction target), and in 2023, although mortality was concentrated in people ≥15 years, it also affected children under 15 (191,000 deaths) ^1^. Pre-existing conditions (e.g., human immunodeficiency virus [HIV] infection, malnutrition, and diabetes mellitus) and other social determinants (e.g., drug dependency and homelessness) increase the risk of treatment failure and raise mortality ^2^. In Colombia, the incidence rate of pulmonary TB for 2023 was 30.65/100,000 inhabitants, while for Cali it was 61.17/100,000 inhabitants, and the trend is increasing as a consequence of missed detection opportunities during COVID-19 ^1^^,^^3^.

Although the burden of TB has been studied in terms of incidence and mortality, studies on years of potential life lost (YPLL) are limited in the Americas. This indicator allows for the quantification of the impact of premature mortality, measuring the years not lived with respect to life expectancy, and provides a more comprehensive view of the social and economic impact of the disease, useful for guiding public policies, prioritizing interventions, and evaluating the effectiveness of control programs ^4^^,^^5^.

Given that mortality in drug-susceptible pulmonary TB is highly preventable with timely standard treatment ^6^, in this study, we estimate the YPLL it generated in Cali, Colombia, between 2009 and 2020.

## THE STUDY

### Design and study population

Operational research through a cross-sectional descriptive study with an analytical approach. We included records of people of any age with a confirmed diagnosis of TB by bacteriological criteria and whose discharge status was classified as deceased due to susceptible TB (defined as cases confirmed of TB by liquid culture, smear microscopy, and/or molecular test, reported as deceased due to TB or TB/HIV coinfection, and who had no finding of drug resistance in initial or follow-up tests).

We excluded records of extrapulmonary TB, drug-resistant TB (≥1 medication), and records without complete information for the outcome variable. Drug-resistant TB, which is monitored and managed differently in Colombia due to its therapeutic complexity and higher mortality, was not included, as this study focuses on deaths from susceptible TB (highly preventable through standard treatment).

### Sources of Information

The District Health Secretariat of Cali provided the database, which is fed by records from the Patient Logbook and the individual treatment card of the Mycobacteria program.

### Variables

We obtained demographic data (sex, age, commune of residence, type of health insurance); clinical data (pre-existing conditions, results of smear microscopy and liquid culture at admission, HIV diagnosis (previous or de novo), and whether they received antiretroviral treatment and/or trimethoprim-sulfamethoxazole); and programmatic data: date of entry into the Mycobacteria program; admission status (new case, previously unfinished treatment due to default, relapse, therapeutic failure, or referred from another program); and date of death. For records with a missing date of death, the age at program entry was used to avoid the loss of 15% of the collected data, given that most people who die from TB do so within the first two months of treatment.

### Statistical analysis

All information was collected in Microsoft 365 Excel sheets and analyzed using Stata 16 TM (Stata Corp, College Station, TX, USA). The population characteristics were summarized using descriptive statistics; numerical variables were checked for normal or non-normal distribution using the Shapiro-Wilk test, with p-values <0.05 considered significant. Numerical variables were summarized as medians and interquartile ranges (IQR), while qualitative variables were summarized as proportions and presented as percentages.

The number of YPLL was calculated by sex, five-year age group, and year of death, using the methods of Romeder and McWhinnie ^4^: we grouped the deaths into five-year intervals, assigned the mean age to each interval, multiplied the death count by the difference between life expectancy and that mean age, and summed the products of all five-year groups. In contrast to the original method (fixed cutoff of 70 years), we used sex- and year-specific life expectancy.

To obtain the standardized YPLL rate, we divided the total YPLL of each group (sex, five-year age group, and year) by the population of Cali of the same sex and age (DANE projections 2009-2020) and then applied age standardization using the direct rate method to compare between populations with different age structures. In cases where the age exceeded the sex- and year-specific life expectancy, those deaths were not included in the YPLL calculation, thus avoiding an overestimation of mortality. Furthermore, the medians of YPLL were compared between the groups defined by each variable, using the Mann-Whitney U test for the comparison of two independent groups and the Kruskal-Wallis test for the comparison between medians of ≥3 groups. A p-value <0.05 was considered significant.

### Ethical aspects

This study was approved by the Research Committee of the Universidad Libre, Cali section (Act No. 009). The data transfer was authorized by the Mycobacteria program of the Cali Health Secretariat.

## FINDINGS

We reviewed 717 records of individuals who died from susceptible TB. One record was excluded due to a reported non-tuberculous mycobacterial infection, and 151 (21.1%) were excluded for being extrapulmonary TB. In total, 565 (78.9%) records were analyzed; 71.5% (404/565) were men, and the median age at death was 56 years (IQR: 38-70).

**Figure 1:**
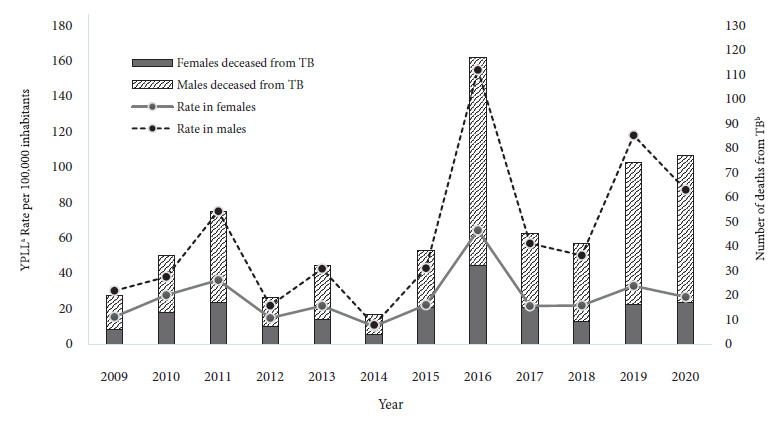
Years of potential life lost from patients who died from sensitive pulmonary tuberculosis in Cali, Colombia, 2009-2020.

^a^ YPLL: years of potential life lost; ^b^ TB: tuberculosis.

**Figure 2:**
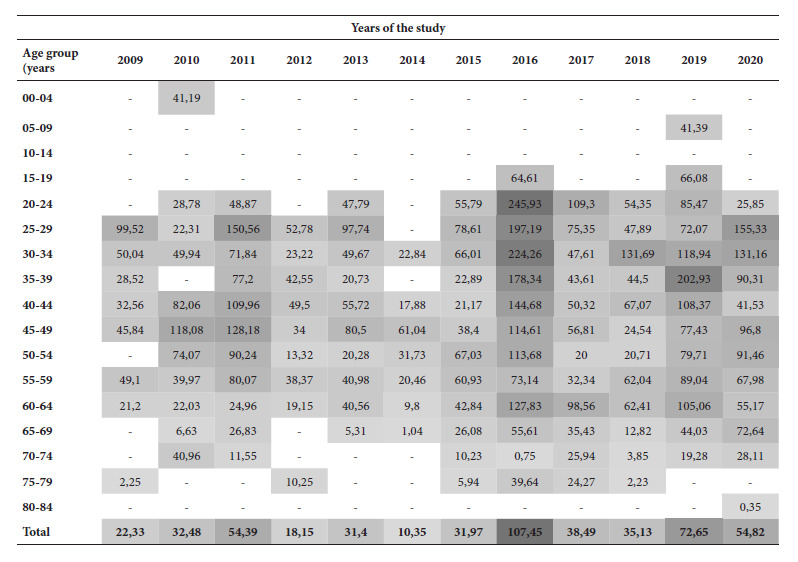
Rate of years of potential life lost per 100,000 inhabitants adjusted by year and age at death from records of patients deceased with susceptible pulmonary tuberculosis in Cali, Colombia, 2009-2020.

The YPLL rate fluctuated several times during the study period, with a peak in 2016 of 154.87/100,000 and 65.62/100,000 inhabitants for men and women, respectively (Figure 1). For the same year, the 20-24 age range had the highest YPLL rate at 245.93/100,000 inhabitants (Figure 2). In total, 11,328 years were lost (median: 17.30 YPLL; IQR: 2.40-34). 79.8% (451/565) of the individuals lost ≥1 year of potential life.

18.8% (90/477) had some vulnerability; of these, 77.7% (70/90) were homeless, 7.7% (7/90) were disabled, 2.2% (2/90) were migrants or displaced persons, and 12.2% (11/90) were from the prison population. 72.3% (285/394) had some pre-existing condition: 38.9% (111/285) malnutrition, 9.1% (26/285) diabetes mellitus, 3.1% (9/285) COPD, and 33.4% (140/418) were people living with HIV (Table 1).

**Table 1:** Bivariate analysis to identify differences between the medians of years of potential life lost by groups from the records of individuals who died prematurely from pulmonary tuberculosis in Santiago de Cali, Colombia 2009-2020.

Variables			n	Total YPLL^a^	Median of total YPLL	IQR^b^	p-value
Demographic factors							
	Ethnicity, n=565						
		Afro-Colombian	57	1220.70	20.90	1.1 - 39.0	0.645^f^
		Mestizo	508	10017.30	17.10	2.7 - 33.9	
	Sex, n=565						
		Male	404	7527.20	15.40	2.0 - 33.6	0.019^f^
		Female	161	3710.80	22.20	4.7 - 35.0	
	Social security scheme, n=565						
		Contributory	178	2659.90	6.30	0 - 28.7	<0.001^g^
		Special	19	428.60	20.30	5.1 - 38.2	
		Uninsured	85	2333.40	28.10	15.5 - 41.4	
		Subsidized	283	5816.10	19.10	5.2 - 34.4	
	Social vulnerability, n=477						
		Yes	90	2500,50	28,50	11.1 - 42.1	<0.001^f^
		No	387	7247,10	16.10	0.9 - 32.7	
Clinical characteristics							
	Malnutrition, n=285						
		Yes	111	2262.80	19.10	4.0 - 34.0	0.805^f^
		No	174	3655.10	19.10	5.2 - 36.1	
	Other pre-existing conditions, n=394						
		Yes	285	5917.90	19.10	5.1 - 34.4	0.405^f^
		No	109	2424.20	23.20	6.7 - 34.5	
	Admission culture for TB^c^, n=198						
		Positive	103	2157.10	21.00	2.4 - 34.0	0.184^f^
		Negative	95	1674.30	13.80	0 - 33.0	
	Admission sputum smear microscopy for TB, n=517						
		Positive	346	7294.60	19.80	3.4 - 35.7	0.137^f^
		Negative	171	3165.50	16.10	2.4 - 30.2	
	De novo HIV^d^, n=418						
		Positive	140	4499.30	33.70	23.3 - 42.5	<0.001^f^
		Negative	278	4549.20	10.60	0 - 28.7	
	Previous HIV, n=140						
		Previous	63	2087.80	34.00	24.4 - 43.2	0.535^f^
		New	77	2411.50	33.20	22.7 - 42.4	
	Received antiretroviral therapy, n=169						
		Yes	59	1776.20	30.80	22.4 - 41.1	0.201^f^
		No	110	2980.40	27.70	13.8 - 40.4	
	Received trimethoprim-sulfamethoxazole, n=112						
		Yes	67	2104.30	32.40	24.7 - 41.4	0.896^f^
		No	45	1454.10	34.00	21.4 - 42.4	
Programmatic factors							
	Admission condition, n=565						
		New	472	9289.10	16.80	2.3 - 33.9	0.029^g^
		Relapse	36	747.60	20.50	3.2 - 34.8	
		Default	33	892.40	25.40	17 - 42.1	
		Failure	6	92.20	11.70	7.1 - 17.3	
		Referred^e^	18	216.70	8.20	0 - 20.0	
	Risk classification for loss to follow-up, n=111						
		Low risk	20	395.50	17.60	1.1 - 33.1	0.889^f^
Intermediate-high risk	91	1726.90	14.00	4.7 - 33.8

a YPLL: years of potential life lost; ^b^ IQR: Interquartile Range; ^c^ TB: tuberculosis; ^d^ HIV: human immunodeficiency virus; ^e^ Referred: referred to a TB program in another city; ^f^ Mann-Whitney U; ^g^ Kruskal-Wallis.

The median YPLL was higher in women (p=0.019), in those without health insurance and those with subsidized and special insurance (p<0.001), in people with social vulnerability (p<0.001), in TB/HIV coinfection (p<0.001), and with an admission status of default from a previous treatment and relapse (p=0.029) (Table 1).

## DISCUSSION

Over a span of 11 years, 565 deaths and 11,328 YPLL due to drug-susceptible pulmonary TB were recorded in a city with an intermediate-to-high incidence of TB in the Americas. The deaths were concentrated among men; ages between 38 and 70 years; the group with comorbidities; with TB/HIV coinfection; and those with an intermediate-to-high risk of being lost to follow-up. The highest median YPLL were observed in those with TB/HIV coinfection, social vulnerability, without health insurance or enrolled in the subsidized or special health regimen, in women, and in those who re-entered after defaulting from previous treatment or due to TB relapse.

YPLL vary according to the study site, methods, and follow-up period. In Pereira, there were 3,753 YPLL between 2010-2015 ^7^, compared to Cali, which had 3,894 YPLL for the same period. YPLL have also been reported in other contexts ^5^^,^^8^, but reports on the subject are limited despite its importance for decision-makers, risk communication, and identifying opportunities for improvement, considering that differences in YPLL also reflect variability in access to health services and in the implementation of care protocols between different contexts ^9^^-^^11^.

Similar to Hoger et al. ^5^, we observed that TB/HIV coinfection is a relevant factor in YPLL. In people living with HIV, it is essential to improve the early detection of active TB (through rapid molecular tests for any suggestive symptom, even if it has been present for <2 weeks) and clinical evaluation for TB prophylaxis ^12^. When TB and HIV are diagnosed concomitantly, it is a priority to ensure medical evaluations and diagnostic tests that allow for the timely initiation of treatment for both infections ^13^. We observed that of the people with TB/HIV coinfection, only 34.9% received antiretroviral therapy. Factors such as social vulnerability and administrative barriers to access could be hindering both diagnosis and timely treatment ^11^^,^^14^^-^^17^. It is a priority to strengthen and facilitate the care pathways for people with this coinfection to ensure timely access to required services.

Timely access does not depend exclusively on insurance coverage ^17^. We observed that in addition to people with social vulnerability and the uninsured, those affiliated with the subsidized and special health regimen also had higher median YPLL compared to those in the contributory regimen. Barriers to achieving effective access to health services, even when they are free, can hinder timely diagnosis and treatment ^11^^,^^14^^,^^16^^-^^19^. Actions aimed at improving and facilitating access to health services are necessary.

Those who were admitted due to default from a previous treatment or TB relapse, and those with social vulnerability also had higher YPLL. These findings are consistent with previous studies that reported that being homeless, being a drug user, having TB/HIV coinfection, as well as having received previous treatment for TB but not completing it for any reason, is associated with death from TB or loss to follow-up ^2^. Since TB is one of the main causes of premature death associated with inequality ^20^, it is crucial to implement strategies that address social determinants and promote both early detection and prioritized multidisciplinary management in critical areas ^15^^,^^20^.

Men accumulated more YPLL in absolute values (7,527.2 vs. 3,710.8), similar to what has been reported in other studies ^7^^,^^8^, but the median YPLL in this study was higher in women (22.2 vs. 15.4), likely due to their higher life expectancy, but a later diagnosis has also been reported ^9^. This finding was consistent with that reported by Hoger et al. ^5^ when they reported 5.11 YPLL in women compared to 4.74 YPLL in men. It is recommended to strengthen Primary Health Care with a gender perspective and active case-finding strategies ^9^.

This study provides important information in a field with limited research, identifies opportunities to design and implement focused strategies for priority groups, and guides public health interventions. We recommend interpreting the results with caution, considering the operational nature and that the data source did not come from the deceased registry. Furthermore, because this study did not analyze YPLL for drug-resistant and extrapulmonary TB—which could underestimate the total burden of YPLL from TB—additional studies are required.

In conclusion, over an 11-year period, 565 deaths and 11,328 YPLL from drug-susceptible pulmonary TB were recorded. The highest median YPLL were observed in those with TB/HIV coinfection, social vulnerability, without health insurance or enrolled in the subsidized or special health regimen, in women, and in those who re-entered after defaulting from previous treatment or due to TB relapse. We recommend the implementation of strategies that manage social determinants, ensure early detection, effective access to health services, active case finding, and timely treatment for both TB and HIV.
