# Supplementary material for: Years of potential life lost due to drug-susceptible pulmonary tuberculosis in Cali, Colombia, 2009-2020
Source: Rev Peru Med Exp Salud Publica. 2025 Sep 8;42(3):300–6. doi: 10.17843/rpmesp.2025.423.14207 (PMC12679976; doi:10.17843/rpmesp.2025.423.14207)
Supplement: Translation

# Translation: Años de vida potenciales perdidos por tuberculosis pulmonar sensible en Cali, Colombia, 2009-2020

**Authors:** Adriana M. Rivas-Mina, Jose F. Fuertes-Bucheli, Jennifer Lozano-García, Laura D. Luna, Yessenia Niño-Ramiréz, Lucy Luna, Robinson Pacheco López

**Affiliations:** 1 Grupo Interdisciplinario de Epidemiología y Salud Pública, Universidad Libre, Cali, Colombia.; 2 Facultad de Ciencias de la Salud, Universidad Icesi, Cali, Colombia.; 3 Secretaría Distrital de Santiago de Cali, Cali, Colombia.

**Keywords:** Esperanza de vida, tuberculosis pulmonar, mortalidad, mortalidad prematura, equidad en salud

## Abstract

Se realizó un estudio descriptivo transversal y analítico que evaluó los años de vida potencialmente perdidos (AVPP) por tuberculosis pulmonar sensible en Cali, Colombia entre 2009 y 2020, usando registros de defunciones de la Secretaría de Salud. Se calcularon los AVPP totales y su tasa anual según edad de muerte y se compararon las medianas de AVPP entre subgrupos. Se analizaron 565 registros de defunciones (71,5% hombres; mediana de edad de 56 años, rango: 38-70). Se generaron 11,238 AVPP en total. Las medianas más elevadas de AVPP se observaron en personas que viven con VIH, en situación de vulnerabilidad social, sin aseguramiento o afiliadas al régimen subsidiado o especial, en mujeres y en quienes reingresaron tras un abandono previo del tratamiento o por recaída de tuberculosis.

## INTRODUCCIÓN

La tuberculosis (TB) es la principal causa de muerte atribuible a un agente infeccioso, con >1 millón de muertes anuales. Entre 2015-2023, la mortalidad disminuyó 23% (lejos de la meta de reducción del 75%) y en 2023, aunque la mortalidad se concentró en personas ≥15 años, afectó también a menores de 15 años (191,000 muertes) ^1^. Enfermedades preexistentes (ej. infección por el virus de la inmunodeficiencia humana [VIH], desnutrición y diabetes mellitus) y otros determinantes sociales (ej. dependencia a las drogas y ser habitante de calle), incrementan el riesgo de no lograr el éxito al tratamiento y aumentan la mortalidad ^2^. En Colombia, la tasa de incidencia de TB pulmonar para 2023 fue de 30,65/100,000 habitantes, mientras que para Cali fue de 61,17/100,000 habitantes, y la tendencia está al aumento como consecuencia de las oportunidades perdidas de detección durante la COVID-19 ^1^^,^^3^.

Aunque se ha estudiado la carga de TB en términos de incidencia y mortalidad, estudios sobre los años de vida potenciales perdidos (AVPP) es limitada en las Américas. Este indicador permite cuantificar el impacto de la mortalidad prematura, midiendo los años no vividos respecto a la esperanza de vida y proporciona una visión más integral del impacto social y económico de la enfermedad, útil para orientar políticas públicas, priorizar intervenciones y evaluar la efectividad de los programas de control ^4^^,^^5^.

Dado que la mortalidad en la TB pulmonar sensible a fármacos es altamente prevenible con tratamiento estándar oportuno ^(^^6^, en este estudio estimamos los AVPP que generó en Cali, Colombia, entre 2009 y 2020.

## EL ESTUDIO

### Diseño y población de estudio

Investigación operativa mediante un estudio descriptivo transversal con enfoque analítico. Incluimos registros de personas de cualquier edad con diagnóstico confirmado de TB mediante criterio bacteriológico y que en su condición de egreso fueron clasificados como fallecidos por TB sensible (definidos como casos confirmados de TB por cultivo líquido, baciloscopia y/o prueba molecular, reportados como fallecido a causa de TB o por coinfección TB/VIH, y que en las pruebas iniciales o de seguimiento no tuvieron hallazgo de farmacorresistencia).

Excluimos registros de TB extrapulmonar, TB farmacorresistente (≥1 medicamento) y registros sin información completa para la variable de desenlace. La TB farmacorresistente, vigilada y manejada de forma diferenciada en Colombia debido a su complejidad terapéutica y mayor mortalidad, no fue incluida, dado que este estudio se enfoca en muertes por TB sensible (altamente prevenibles mediante el tratamiento estándar).

### Fuentes de la Información

La Secretaría de Distrital de Salud de Cali suministró la base de datos, la cual es alimentada por los registros del Libro de Pacientes y la tarjeta individual de tratamiento del programa de Micobacterias.

### Variables

Obtuvimos datos demográficos (sexo, edad, comuna de residencia, tipo de aseguramiento en salud); datos clínicos condiciones preexistentes, resultado de baciloscopia y cultivo líquido al ingreso, diagnóstico de VIH (previo o de novo), y si recibió tratamiento antirretroviral y/o trimetoprima-sulfametoxazol); y datos programáticos: fecha de ingreso al programa de Micobacterias; condición de ingreso (caso nuevo, tratamiento previo no terminado por abandono, recaída, fracaso terapéutico o remitido de otro programa); y fecha de fallecimiento. Para los registros con fecha de muerte faltante se usó la edad de ingreso al programa para evitar la pérdida del 15% de los datos recolectados, dado que la mayoría de personas que fallecen por TB lo hacen durante los dos primeros meses del tratamiento.

### Análisis estadístico

Toda la información fue recolectada en hojas de Excel de Microsoft 365 y analizada usando Stata 16 TM (Stata Corp, Collage Station, TX, USA). Las características de la población fueron resumidas mediante estadística descriptiva; a las variables numéricas se les revisó si su distribución era normal o no, a través de Shapiro-Wilk; considerando significantes los *p*-valores <0,05. Las variables numéricas se resumieron en medianas y rangos intercuartílicos (RIC), mientras que las variables cualitativas se resumieron como proporciones y se presentaron en porcentajes.

Se calculó el número de AVPP por sexo, quinquenio de edad y año de fallecimiento, usando los métodos de Romeder y McWhinnie ^4^: agrupamos las defunciones en intervalos de cinco años, asignamos a cada intervalo la edad media, multiplicamos el conteo de defunciones por la diferencia entre la esperanza de vida y esa edad media, y sumamos los productos de todos los quinquenios. En contraste con el método original (corte fijo 70 años), usamos la esperanza de vida específica por sexo y año.

Para obtener la tasa estandarizada de AVPP, dividimos el total de AVPP de cada grupo (sexo, quinquenio y año) por la población de Cali de ese mismo sexo y edad (proyecciones DANE 2009-2020) y luego aplicamos la estandarización por edad mediante el método de tasas directas para comparar entre poblaciones con distintas estructuras etarias. En los casos en que la edad superó la esperanza de vida específica por sexo y año, dichas defunciones no se tuvieron en cuenta en el cálculo en el número de AVPP, evitando así sobreestimar la mortalidad. Además, se comparó las medianas de los AVPP entre los grupos definidos por cada variable, utilizando U-Mann-Whitney para la comparación de dos grupos independientes y Kruskal Wallis para la comparación entre las medianas de ≥3 grupos. Un p-valor <0,05 fue considerado significativo.

### Aspectos éticos

Este estudio fue aprobado por el Comité de Investigación de la Universidad Libre seccional Cali (Acta N°: 009). La trasferencia de datos fue autorizada por el programa de Micobacterias de la Secretaría de Salud de Cali.

## HALLAZGOS

Se revisaron 717 registros de personas fallecidas por TB sensible. Se excluyó un registro con reporte de infección por micobacteria no tuberculosa, y 151 (21,1%) por ser TB extrapulmonar. En total, se analizaron 565 (78,9%) registros, 71,5% (404/565) fueron hombres y la mediana de edad al fallecer fue de 56 años (RIC: 38-70).

La tasa de AVPP tuvo varias oscilaciones durante el periodo de estudio con un pico en 2016 de 154,87/100,000 y 65,62/100,000 habitantes para hombres y mujeres, respectivamente (Figura 1) y para el mismo año, el rango de edad entre 20 y 24 años tuvo la mayor tasa de AVPP con 245,93/100,000 habitantes (Figura 2). En total se perdieron 11,328 años, (mediana: 17,30 AVPP; RIC: 2,40-34). El 79,8% (451/565) de las personas perdieron ≥1 año de vida potencial.

**Figura 1:** Años de vida potencialmente perdidos de los pacientes fallecidos por tuberculosis pulmonar sensible en Cali, Colombia, 2009-2020.

^a^ AVPP: años de vida potencialmente perdidos; ^b^ TB: tuberculosis.

**Figura 2:** Tasa de años de vida potencialmente perdidos por 100,000 habitantes ajustada por año y edad de muerte de registros de pacientes fallecidos con tuberculosis pulmonar sensible en Cali, Colombia, 2009-2020.

El 18,8% (90/477) tenían alguna vulnerabilidad; de estos, 77,7% (70/90) eran personas en situación de calle, 7,7% (7/90) discapacitados, 2,2% (2/90) migrantes o desplazados, y 12,2% (11/90) población privada de la libertad. El 72,3% (285/394) presentó alguna condición preexistente: 38,9% (111/285) desnutrición, 9,1% (26/285) diabetes mellitus, 3,1% (9/285) EPOC y 33,4% (140/418) eran personas que viven con VIH (Tabla 1).

**Tabla 1:** Análisis bivariado para identificar diferencias entre las medianas de años de vida potencialmente perdidos por grupos de los registros de personas fallecidos prematuramente con tuberculosis pulmonar en Santiago de Cali, Colombia 2009-2020.

Variables			n	AVPP^a^ total	Mediana de AVPP total	RIC^b^	Valor p
Factores demográficos							
	Etnia, n=565						
		Afrocolombiano	57	1220,70	20,90	1,1 - 39,0	0,645^f^
		Mestizo	508	10017,30	17,10	2,7 - 33,9	
	Sexo, n=565						
		Hombre	404	7527,20	15,40	2,0 - 33,6	0,019^f^
		Mujer	161	3710,80	22,20	4,7 - 35,0	
	Régimen de seguridad social, n=565						
		Contributivo	178	2659,90	6,30	0 - 28,7	<0,001^g^
		Especial	19	428,60	20,30	5,1 - 38,2	
		No asegurado	85	2333,40	28,10	15,5 - 41,4	
		Subsidiado	283	5816,10	19,10	5,2 - 34,4	
	Vulnerabilidad social, n=477						
		Si	90	2500,50	28,50	11,1 - 42,1	<0.001^f^
		No	387	7247,10	16,10	0,9 - 32,7	
Características clínicas							
	Desnutrición, n=285						
		Si	111	2262,80	19,10	4,0 - 34,0	0,805^f^
		No	174	3655,10	19,10	5,2 - 36,1	
	Otras condiciones preexistentes, n=394						
		Si	285	5917,90	19,10	5,1 - 34,4	0,405^f^
		No	109	2424,20	23,20	6,7 - 34,5	
	Cultivo para TB^c^ de ingreso, n=198						
		Positivo	103	2157,10	21,00	2,4 - 34,0	0,184^f^
		Negativo	95	1674,30	13,80	0 - 33,0	
	Baciloscopia para TB de ingreso, n=517						
		Positivo	346	7294,60	19,80	3,4 - 35,7	0,137^f^
		Negativo	171	3165,50	16,10	2,4 - 30,2	
	VIH^d^ de novo, n=418						
		Positivo	140	4499,30	33,70	23,3 - 42,5	<0,001^f^
		Negativo	278	4549,20	10,60	0 - 28,7	
	VIH previo, n=140						
		Previo	63	2087,80	34,00	24,4 - 43,2	0,535^f^
		Novo	77	2411,50	33,20	22,7 - 42,4	
	Recibió terapia antirretroviral, n=169						
		Si	59	1776,20	30,80	22,4 - 41,1	0,201^f^
		No	110	2980,40	27,70	13,8 - 40,4	
	Recibió trimetoprima-sulfametoxazol, n=112						
		Si	67	2104,30	32,40	24,7 - 41,4	0,896^f^
		No	45	1454,10	34,00	21,4 - 42,4	
Factores programáticos							
	Condición de ingreso, n=565						
		Nuevo	472	9289,10	16,80	2,3 - 33,9	0,029^g^
		Recaída	36	747,60	20,50	3,2 - 34,8	
		Abandono	33	892,40	25,40	17 - 42,1	
		Fracaso	6	92,20	11,70	7,1 - 17,3	
		Remitido^e^	18	216,70	8,20	0 - 20,0	
	Clasificación riesgo de pérdida de seguimiento, n=111						
		Riesgo bajo	20	395,50	17,60	1,1 - 33,1	0,889^f^
		Riesgo intermedio-alto	91	1726,90	14,00	4,7 - 33,8	

a AVPP: años de vida potencialmente perdidos; ^b^ RIC: Rango Intercuartílico; ^c^ TB: tuberculosis; ^d^ VIH: virus de la inmunodeficiencia humana; ^e^ Remitido: referido a un programa de TB de otra ciudad; ^f^ U de Mann-Whitney; ^g^ Kruskal-Wallis.

La mediana de AVPP fue mayor en mujeres (p=0,019), en no asegurados en salud y aquellos con aseguramiento subsidiado y especial (p<0,001), en personas con vulnerabilidad social (p<0,001), en coinfección TB/VIH (p<0,001), y con condición de ingreso como abandono a un tratamiento previo y recaída (p=0,029) (Tabla 1).

## DISCUSIÓN

En un lapso de 11 años se registraron 565 muertes y 11,328 AVPP por TB pulmonar sensible en una ciudad de intermedia-alta incidencia de TB en las Américas. Las defunciones se agruparon en hombres; edades entre 38 y 70 años; el grupo con comorbilidades; con coinfección TB/VIH; y aquellos con riesgo intermedio-alto de pérdida en el seguimiento. Las medianas de AVPP más elevadas se observaron en aquellos con coinfección TB/VIH, vulnerabilidad social, sin aseguramiento en salud o adscritos al régimen de salud subsidiado o especial, en mujeres y en aquellos que reingresaron tras abandono de tratamiento previo o por recaída de TB.

Los AVPP varían de acuerdo con el sitio de estudio, métodos y periodo de seguimiento. En Pereira hubo 3753 AVPP entre 2010-2015 ^7^, en comparación con Cali que para ese mismo periodo tuvo 3894 AVPP. En otros contextos también se ha informado de AVPP ^5^^,^^8^, pero los informes sobre el tema son limitados a pesar de su importancia también para los tomadores de decisiones, la comunicación del riesgo y la identificación de oportunidades de mejora, considerando que las diferencias en AVPP también reflejan variabilidad en el acceso a los servicios de salud y en la implementación de protocolos de atención entre los distintos contextos ^9^^-^^11^.

Al igual que Hoger *et al.*^5^, observamos que la coinfección TB/VIH es un factor relevante en los AVPP. En personas que viven con VIH resulta fundamental mejorar la detección precoz de TB activa (mediante pruebas moleculares rápidas ante cualquier síntoma sugestivo, incluso de <2 semanas de evolución) y la evaluación clínica para profilaxis contra TB ^12^. Cuando se realiza el diagnóstico de TB y VIH de manera concomitante, resulta prioritario garantizar las evaluaciones médicas y pruebas diagnósticas que permitan el inicio oportuno del tratamiento para ambas infecciones ^(^^13^. Observamos que de las personas con coinfección TB/VIH, solo el 34,9% recibió terapia antirretroviral. Factores como la vulnerabilidad social y barreras administrativas de acceso podrían estar dificultando tanto el diagnóstico como el tratamiento oportuno ^(^^11^^,^^14^^-^^17^. Resulta prioritario reforzar y facilitar las rutas de atención de personas con esta coinfección para garantizar el acceso oportuno a los servicios requeridos.

El acceso oportuno no depende exclusivamente del aseguramiento ^(^^17^. Observamos que además de las personas con vulnerabilidad social y aquellos no asegurados, quienes estaban afiliados al régimen de salud subsidiado y especial también presentaron medianas más altas de AVPP en comparación con los afiliados al régimen contributivo. Barreras en la materialización del acceso efectivo a los servicios de salud incluso cuando son gratuitos pueden dificultar el diagnóstico y tratamiento oportuno ^(^^11^^,^^14^^,^^16^^-^^19^. Acciones orientadas a mejorar y facilitar el acceso a los servicios de salud son necesarios.

Quienes ingresaron por abandono a un tratamiento previo o por recaída de TB, y quienes presentaban vulnerabilidad social también presentaron mayores AVPP. Los hallazgos son consistentes con estudios previos que informaron que estar en situación de calle, ser farmacodependiente, tener coinfección de TB/HIV, así como haber recibido tratamiento previo para TB, pero no haberlo terminado por cualquier causa, se asocia con la muerte por TB o la pérdida en el seguimiento ^2^. Debido a que la TB es una de las principales causas de muerte prematura asociada a desigualdad ^20^, resulta fundamental implementar estrategias que aborden los determinantes sociales y fomenten tanto la detección temprana, como el manejo multidisciplinar priorizado en áreas críticas ^(^^15^^,^^20^.

Los hombres acumularon más AVPP en valores absolutos (7 527,2 vs. 3 710,8), similar a lo reportado en otros estudios ^7^^,^^8^, pero la mediana de AVPP en este estudio fue mayor en mujeres (22,2 vs. 15,4), probablemente debido a su mayor esperanza de vida, pero también se ha informado de un diagnóstico más tardío ^9^. Este hallazgo fue consistente con lo informado por Hoger *et al*. ^5^ cuando informaron 5,11 AVPP en mujeres en comparación con 4,74 AVPP en hombres. Se recomienda fortalecer la Atención Primaria en Salud con enfoque de género y estrategias de búsqueda activa de casos ^(^^9^.

Este estudio aporta información importante en un campo con investigaciones limitadas, permite identifica oportunidades para diseñar e implementar estrategias focalizadas en grupos prioritarios y orientar intervenciones en salud pública. Recomendamos interpretar los resultados con cautela, considerando la naturaleza operativa y que la fuente de datos no provino del registro de fallecidos. Además, debido a que en este estudio no se analizaron los AVPP por TB farmacorresistente y extrapulmonar-lo cual podría subestimar la carga total de AVPP por TB-estudios adicionales son requeridos.

En conclusión, durante un periodo de 11 años se registraron 565 muertes y 11,328 años de AVPP por TB pulmonar sensible. Las medianas de AVPP más elevadas se observaron en aquellos con coinfección TB/VIH, vulnerabilidad social, sin aseguramiento en salud o adscritos al régimen de salud subsidiado o especial, en mujeres y en aquellos que reingresaron tras abandono de tratamiento previo o por recaída de TB. Recomendamos la implementación de estrategias que gestionen los determinantes sociales, que garanticen detección temprana, el acceso efectivo a los servicios de salud, la búsqueda activa de casos y el tratamiento oportuno tanto para TB como para VIH.
